# Robust prostate disease classification using transformers with discrete representations

**DOI:** 10.1007/s11548-024-03153-8

**Published:** 2024-05-13

**Authors:** Ainkaran Santhirasekaram, Mathias Winkler, Andrea Rockall, Ben Glocker

**Affiliations:** 1 Department of Computing, Imperial College London, London, UK; 2 Department of Surgery and Cancer, Imperial College London, London, UK

**Keywords:** Biomedical imaging, Robustness, Computer-aided diagnosis, Machine learning, Neural networks

## Abstract

**Purpose:**

Automated prostate disease classification on multi-parametric MRI has recently shown promising results with the use of convolutional neural networks (CNNs). The vision transformer (ViT) is a convolutional free architecture which only exploits the self-attention mechanism and has surpassed CNNs in some natural imaging classification tasks. However, these models are not very robust to textural shifts in the input space. In MRI, we often have to deal with textural shift arising from varying acquisition protocols. Here, we focus on the ability of models to generalise well to new magnet strengths for MRI.

**Method:**

We propose a new framework to improve the robustness of vision transformer-based models for disease classification by constructing discrete representations of the data using vector quantisation. We sample a subset of the discrete representations to form the input into a transformer-based model. We use cross-attention in our transformer model to combine the discrete representations of T2-weighted and apparent diffusion coefficient (ADC) images.

**Results:**

We analyse the robustness of our model by training on a 1.5 T scanner and test on a 3 T scanner and vice versa. Our approach achieves SOTA performance for classification of lesions on prostate MRI and outperforms various other CNN and transformer-based models in terms of robustness to domain shift and perturbations in the input space.

**Conclusion:**

We develop a method to improve the robustness of transformer-based disease classification of prostate lesions on MRI using discrete representations of the T2-weighted and ADC images.

## Introduction

Prostate disease classification on multi-parametric MRI (mpMRI) is performed by radiologists using a standardised reporting lexicon called PI-RADS. However, it is still somewhat subjective and results in a false-positive rate of 30–40 per cent leading to a large number of unnecessary biopsies [1]. Therefore, automated prostate disease classification would be of great benefit to reduce the number of unnecessary biopsies. Automated disease classification on prostate MRI has shifted from hand-crafted radiomics features to CNNs for automated classification. Recently, transformer networks have revolutionised natural language processing (NLP) using self-attention to better model long-range dependencies in the input data to extract more global information [2]. Transformer networks have now been extended to image classification tasks in what is called the vision transformer [3]. However, its performance is hugely dependent on pre-training on large datasets. Furthermore, both CNNs and transformers are susceptible to adversarial attacks and real-world corruptions, raising concerns about their reliability in critical applications like healthcare.

Our focus in this work is on single-domain generalisability (SDG), addressing challenges posed by variations in MRI acquisition and magnet strengths across different sites. As MRI scanners evolve, it is crucial for deep learning models to generalise across new scanners without retraining. We propose an architectural design based on discrete representation learning, inspired by human reasoning with categorical representations [4]. Categorical representations, like stating whether a lesion is dark or bright, allow robust classification by ignoring noise or textural shifts. In the context of prostate MRI, our approach aims to enhance disease classification robustness under acquisition shifts from 1.5 T to 3 T scanners and vice versa.

## Related works

It was shown that model accuracy is not maintained under different types of noise and distortions, suggesting poor model robustness [5]. To enhance model robustness, common techniques include data augmentation, adversarial training, and architecture design.

In data augmentation, we enhance model robustness by synthesising new training data from the existing training set to encourage model invariance to various perturbations. It was shown that model accuracy is not maintained under different types of noise and distortions [5]. Therefore, augmenting training data with specific corruptions helps preserve accuracy for those corruptions, but not for unseen ones [5]. In medical imaging, BigAug is an aggressive augmentation scheme which generates training data with a series of augmentation techniques which significantly improve segmentation performance in the SDG setting [6]. It was highlighted by [5] that neural networks overly rely on texture information. This is in contrast with humans who prefer shape for classification. Therefore, style transfer was used by [5] to augment ImageNet with textured variants. They achieved improved accuracy on unseen common perturbations in ImageNet-C and provided evidence that this increases the shape bias of CNNs. Similarly, RandConv [7] proposes using randomised convolutions in the initial layers of CNNs to extract shape-biased features. Recently, there have been augmentation strategies which force neural networks to learn even more generalisable features such as CutOut [8] and MixUp [9]. In CutOut [8], random patches of an image are cropped out which encourages more global feature learning for classification. MixUp [9] linearly combines randomly sampled training images and labels which leads to more stable predictions on data sampled outside the training distribution such as those degraded by artefacts.

SMOTE, which stands for Synthetic Minority Oversampling Technique, is a method used to balance class distribution within a dataset through oversampling [10]. This technique involves identifying minority examples in close proximity within the feature space. Subsequently, it establishes a line between these examples in the feature space and generates new samples at points along that line. Since the advent of SMOTE, there have been a significant number of extensions of SMOTE [11]. For example, the initial enhancements to SMOTE aimed to address its known limitations of generating overlapping and noisy examples. This was achieved by adding a noise filtering step immediately after the SMOTE process. Two common techniques for this purpose are SMOTE-TomekLinks and SMOTE+ENN, as proposed by [12]. Filtering out artificial examples is a crucial operation that contributes to the effectiveness of SMOTE, particularly when dealing with real-world data. SMOTE has also a successful impact in medical imaging such as in the accurate prediction of COVID-19 on chest x-rays [13] and improving the AUC score for glioblastoma patient survival prediction [14].

**Fig. 1:**
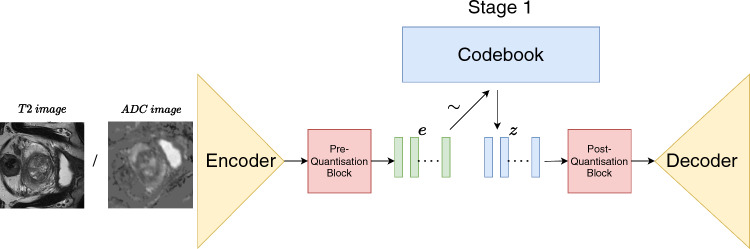
First stage of training. We learn discrete representations in the form of a codebook for T2-weighted and ADC images

Adversarial training schemes akin to data augmentation methods also manipulate the input data but with a specific objective. In methods such as [15], a min-max problem is constructed in which the inner maximisation seeks effective perturbations from a distribution such as Gaussian noise, while the outer minimisation updates the model parameters to minimise expected error. In an extension of this method, [16] learns the noise distribution from which to sample the perturbations. There have since been more sophisticated adversarial training schemes developed. For example, M-ADA [17] introduces adversarial data augmentation through a meta-learning approach, utilising a Wasserstein auto-encoder to create new domains. In medical imaging, AdvBias [18] develops an adversarial training scheme specific to MRI data. Here, they employ adversarial data augmentation in the input space to learn the generation of bias field deformations.

In concurrent research, [19] adopts an architectural design approach identical to us to address the issue of the vision transformer not generalising well to out-of-distribution, real-world data. They proposed a simple yet effective modification to the vision transformer’s input layer by incorporating discrete tokens generated by a vector-quantised encoder. They demonstrate that this approach makes the vision transformer less sensitive to perturbations in the input space and also showed up to a 12% robustness improvement in terms of the relative corruption error across seven ImageNet benchmarks for four architecture variants.

## Method

Our method is divided into two stages. The first stage aims to learn a discrete representation of both the T2-weighted and ADC images. The second stage then utilises the T2-weighted and ADC low-dimensional discrete representations as input into a transformer- based architecture to predict the disease class of the prostate lesion.

### Stage 1

The first stage of our method is learning discrete representations for both the T2-weighted and ADC images as shown in Fig. 1.

We use the VQ-VAE [20]. In this, the discrete latent space is a categorical distribution defined as a codebook, $\mathbb {D} \in \mathbb {R}^{K\times d}$ where *K* is the number of elements in the codebook and *d* is the dimensionality of each vector in the codebook. We denote the $j^{th}$ element in $\mathbb {D}$ as $l_j$.

The VQ-VAE firstly consists of an encoder $\phi _e$ which maps the inputs space, *x* to the continuous latent vectors, *e* which are then discretised using vector quantisation to form discrete variables, $\hat{z}$ as visualised in Fig. 1. The decoder, $\phi _d$, maps the discrete latent vectors to the output space, *y*. The quantisation of the continuous space is performed by firstly dividing *e* into *m* vectors. We spatially divide *e* the continuous latent space of size, $c \times x \times y \times z$ into vectors, $e_i$ of size $c \times 1 \times 1 \times 1$ where *c* is the number of channels. We then replace $\forall e_i \in e$ with the nearest element in $l_k \in \mathbb {D}$ sampled by euclidean distance to form the discrete latent variables, $\hat{z}$, where $k=argmin_j\Vert e_i - l_j\Vert _2$.

1 $$\begin{aligned}{} & {} q(\hat{z}_i = l_j\mid x) \nonumber \\{} & {} = \left\{ \begin{array}{ll} 1 \qquad \text {for} \quad l_k \in \mathbb {D}, k = \arg \min _j\Vert e_i - l_j\Vert _2\\ 0 \qquad \text {otherwise} \end{array} \right\} \end{aligned}$$

We cannot backpropogate through this sampling operation to update the codebook and therefore approximate the gradients for $\mathbb {D}$ using straight-through gradient approximation. This is achieved by copying the gradients from the decoder input, *z*, to the encoder output, *e*. In order to learn the codebook, we move $l_k$ closer to $e_i$ by euclidean distance. This is captured in the second term of the loss function shown in Eq. (2) where a stop gradient (sg) is applied to $e_i$ which sets the gradient attached to $e_i$ to 0 and constrains $e_i$ to a non-updated constant. The volume of the continuous embedding space can grow arbitrarily large during training, and therefore, a commitment loss is applied shown in the third term in Eq. (2). The first terms in Eq. (2) are the reconstruction loss term computed using the mean square error. We use a $\beta$ value of 0.25 as suggested in [20]. The codebook elements are initialised uniformly from $-1/K$ to 1/*K*.

2 $$\begin{aligned} \mathcal {L} ={} & {} \log p(x | \hat{z}) +\sum _{i=0}^{i=m}\Vert sg(e_i)-l_k\Vert _2 \nonumber \\{} & {} + \sum _{i=0}^{i=m} \beta \Vert e_i-sg(l_k)\Vert _2 \ \end{aligned}$$

### Multi-headed self-attention (MHSA)

We first describe the MHSA mechanism required to understand stage 2 of our method. The first stage of the MHSA is to convert each input vector into a *d*-dimensional query (*q*), key (*k*) and value (*v*) vector with a linear layer, which are then concatenated, respectively, over all input vectors to form the *Q*, *K* and *V* matrices. The general equation for self-attention is shown in Eq. (3). Attentions scores between different input vectors are calculated with the dot product between *Q* and *V* to construct the attention matrix, *A*, which is normalised before multiplying with the value matrix *V* to form the output.

3 $$\begin{aligned} \textrm{Output} = \textrm{softmax}\left( \frac{Q.K^T}{\sqrt{d}}\right) \times V \end{aligned}$$

In multi-headed self-attention, one boosts the performance of single-head self-attention by applying multiple attention heads with different learnt *Q*, *K* and *V* matrices for each head.

### Stage 2

**Fig. 2:**
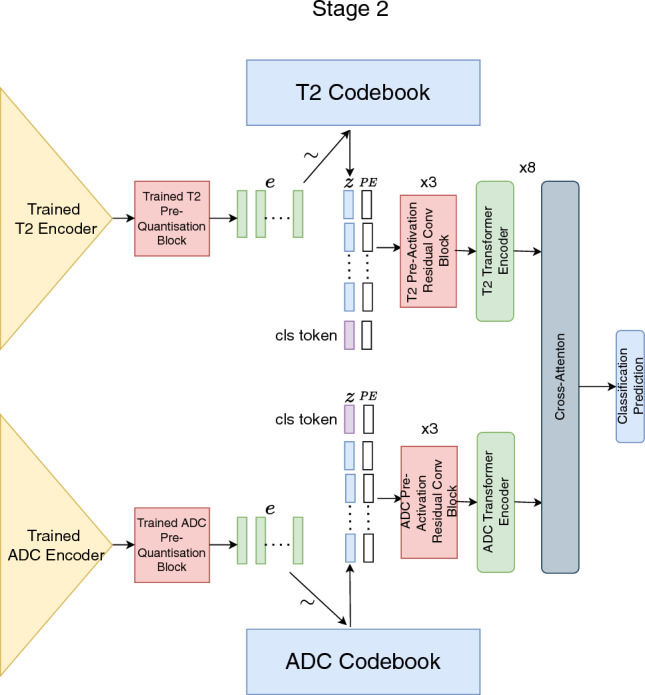
Second stage of training. We train a cross-attention architecture with inputs sampled for the T2 and ADC dictionary

In the second stage of training visualised in Fig. 2, we freeze the T2 encoder, ADC encoder, T2 pre-quantisation block, ADC pre-quantisation block, T2 dictionary and ADC dictionary weights. The T2 and ADC images are passed through their respective encoders before sampling their respective dictionary to form the discrete latent variables, $\hat{z}_{T2} \in \mathbb {R}^{256 \times 16 \times 16 \times 12}$ and $\hat{z}_{ADC} \in \mathbb {R}^{256 \times 16 \times 16 \times 12}$ as shown in Fig. 2. $\hat{z}_{T2}$ and $\hat{z}_{ADC}$ then, respectively, pass through three pre-activation residual convolutional blocks with no weight sharing (Fig. 2). Next, the output of the pre-activation convolutional blocks is divided into *m* tokens similar to quantisation where $m = x \times y \times z$ to produce $\tilde{z}_{T2}$ and $\tilde{z}_{ADC}$. A T2 and ADC class token is concatenated to $\tilde{z}_{T2}$ and $\tilde{z}_{ADC}$, respectively, which are then consumed by the T2 and ADC transformer, respectively. Positional encodings (PE) as performed by [3] are applied to each of tokens. The transformer layer is the same transformer architecture as the vision transformer [3]. However, hereafter each transformer layer we apply a cross-attention layer for the transfer of semantic information between the T2 and ADC transformers.

The cross-attention mechanism [21] we use after the transformer blocks for multi-scale fusion is demonstrated in Fig. 2. Here we propose to first take the output class token from the T2 transformer which we expect to contain all the salient information representative of the T2 image and concatenate with the tokens outputted from the ADC transformer excluding the ADC class token. We apply a linear projection of the T2 class token to form a single query. The keys and values are formed by linear projections of the ADC tokens before passing the query, keys and values through multi-headed self-attention. The process is repeated for the transfer of salient information contained in the ADC class token to the T2 tokens.

Alternate layers of transformer and cross-attention layers are repeated 8 times which allows to distill increasingly abstract knowledge between the tokens of the T2 and ADC transformer. Finally, the class tokens from the T2 and ADC transformers are concatenated before passing through a multi-layer perceptron (MLP) for class prediction highlighted in Fig. 2.

We only optimise the weights in the pre-activation residual convolutional blocks, transformer layers and cross-attention layers while the rest of the network is frozen in stage 2 of our framework. We use the cross-entropy loss function with equal weighting for each class to optimise the trainable weights in stage 2 of our model.

**Table 1:** 1.5 T and 3 T splits showing the number of scans from the internal and external dataset for each risk group

Internal data					
1.5 T			3 T		
Low risk	Medium risk	High risk	Low risk	Medium risk	High risk
40	40	40	0	14	20
External data					
1.5 T			3 T		
Low risk	Medium risk	High risk	Low risk	Medium risk	High risk
0	0	0	40	26	20

**Table 2:** T2- and diffusion-weighted image acquisition parameters for the 1.5 T and 3 T datasets

1.5 T					
T2			ADC		
Slice thickness	Axial resolution	Sequence	Slice thickness	Axial resolution	Sequence
3–4 mm	0.325–0.625 mm	Turbo SE	3.6–4 mm	2–2.5 mm	Single-shot EP
3 T					
T2			ADC		
Slice thickness	Axial resolution	Sequence	Slice thickness	Axial resolution	Sequence
3–3.6 mm	0.325–0.5 mm	Turbo SE	3.6–4 mm	2–2.5 mm	Single-shot EP

ADC maps in both datasets are calculated with b-values; 50, 400 and 800

## Dataset preparation

### Pre-processing and augmentation

All MRI images and their corresponding segmentations were, respectively, re-sampled with cubic B-spline interpolation and nearest-neighbour, respectively, to a resolution of 0.5 mm $\times 0.5$ mm $\times 1.5$ mm to match the anisotropic resolution of the images.

A patch of size of $128 \times 128 \times 8$ centred on the lesion is cropped. We normalise all images by re-scaling the intensities between 0 and 1.

We carry out various spatial transformations to augment the training dataset. This includes vertical or horizontal flipping followed by random rotations between −90 and 90 degrees.

### Dataset

We create two different source domains with an internal dataset created in-house and an external dataset. The external dataset is made up of T2-weighted axial, diffusion weighted imaging (b-800), ADC maps and K-trans image from the ProstateX challenge which were acquired on a 3 Tesla scanner from a single sites [22]. This dataset consists of 330 pre-selected lesions with Gleason score labels. The dataset is highly imbalanced with only 23 per cent of lesions classed as clinically significant (Gleason grade group (GGG) 2 and above).

The internal dataset consists of T2-weighted axial and ADC maps which were acquired on either a 1.5 or 3 Tesla scanners. This dataset is made up of patients all of whom have received radical prostatectomy. We use the histology as ground truth. This dataset consists of 154 lesions from 100 patients. In this dataset, 120 lesion are acquired on a 3 T scanner and 34 lesions are acquired on a 1.5 T scanner. We divide our internal and external dataset into three risk groups based on the Gleason score: low risk—GGG 1, medium risk—GGG 2-3, and high risk—GGG 4-5.

We use our internal and external dataset to extract 40 lesions for each risk group which were acquired on a 1.5 T and 3 T scanner. In the table below, we show 40 lesions acquired from a 1.5 T for each risk group all of which are from the internal dataset. The 3 T dataset shown in Table 1 is acquired from a mixture of our internal and external dataset due to 23% of lesions being clinically significant in the external dataset.

We also show the acquisition parameters for the 1.5 T and 3 T dataset in Table 2. Note also the mild distribution shift in the axial resolution and slice thickness in the T2-weighted images from the 1.5 T to 3 T scanner.

## Experimental setup

### Model

We use an hybrid 2D/3D VQ-VAE in order to handle the anisotropic nature of prostate MRI images. In every layer of the encoder and decoder, we use pre-activation convolutional blocks consisting of leaky ReLU activation and group normalisation (2 groups). Our VQ-VAE consists of 5 levels with the architecture shown in Table 3. Ablation experiments revealed that a minimum of 128 codebook vectors are required to minimise reconstruction error below 0.001 with a mean square error loss. We therefore only use 128 codebook vectors in the codebook dictionary for all experiments

**Table 3:** VQ-VAE architecture

	Encoder			Decoder		
Layer	Convolutions	Downsample	Output ($c\times x\times y\times z$)	Convolutions	Upsample	Output ($c\times x\times y\times z$)
Conv Block 1	$$\begin{bmatrix} 3\times 3\times 1, 32 \\ 3\times 3\times 1, 32 \end{bmatrix}$$ $\times 2$	2D Max-Pooling	$32\times 64\times 64\times 12$	$$\begin{bmatrix} 3\times 3\times 3, 32 \\ 3\times 3\times 3, 256 \end{bmatrix}$$ $\times 2$	Bi-linear	$256\times 32\times 32\times 12$
Conv Block 2	$$\begin{bmatrix} 3\times 3\times 1, 64 \\ 3\times 3\times 1, 64 \end{bmatrix}$$ $\times 2$	2D Max-Pooling	$64\times 32\times 32\times 12$	$$\begin{bmatrix} 3\times 3\times 1, 128 \\ 3\times 3\times 1, 128 \end{bmatrix}$$ $\times 2$	Bi-linear	$64\times 64\times 64\times 12$
Conv Block 3	$$\begin{bmatrix} 3\times 3\times 1, 128 \\ 3\times 3\times 1, 128 \end{bmatrix}$$ $\times 2$	2D Max-Pooling	$128\times 16\times 16\times 12$	$$\begin{bmatrix} 3\times 3\times 1, 64 \\ 3\times 3\times 1, 64 \end{bmatrix}$$ $\times 2$	Bi-linear	$32\times 256\times 256\times 12$
Conv Block 4	$$\begin{bmatrix} 3\times 3\times 3, 256 \\ 3\times 3\times 3, 256 \end{bmatrix}$$ $\times 2$	None	$128\times 16\times 16\times 12$	$$\begin{bmatrix} 3\times 3\times 1, 32 \\ 3\times 3\times 1, 1 \end{bmatrix}$$ $\times 2$	None	$1\times 256\times 256\times 12$

The transformer is the same architecture as the vision transformer [3] consisting of 8 layers and 8 heads in MHSA. The MLP in our transformers model has an input of $1\times 256$ with an expansion ratio of 2. We also use 8 heads in cross-attention.

### Single-domain generalisation experiment

In this set of experiments, we compare our method to the 3D ResNet-50, vision transformer and hybrid vision transformer aided with domain generalisation methods under an acquisition shift. We choose methods which focus on aggressive data augmentation, adversarial learning and self-supervised learning to build more robust representations. Specifically, the SDG methods used are BigAug [6], ProstAdv [23] and Jigen [24]. ProstAdv is an adversarial technique which uses the decoupling direction and norm (DDN) method [25]. DDN produces gradient-oriented adversarial examples that provoke mis-classification with minimal L2 norm variations by decoupling the direction and adding adversarial perturbation to the image. The self-supervised method Jigen [24] is applied to the training set and used to initialise the weights of the classification model. The hybrid vision transformer consists of a modified ResNet-26 followed by a vision transformer made up of 12 layers and 8 heads.

### Perturbation experiments

In the perturbation experiments, we want to remove acquisition shift from training to test in order to assess for only the perturbation effect on classification performance. Therefore, training and testing are all performed on a 3 T scanner. Here, we divided 3 T dataset defined in Tables 1 and 2 such that there are 30 lesions in each risk group in the training set and 10 lesions in each risk group in the test set. We compare our method to the ResNet-50, vision transformer and hybrid vision transformer under various types of perturbations applied to the test set.

We adjust noise levels between 1 and 30 % (1, 5, 10, 15, 20, 25, 30 %) for Gaussian, Poisson and Salt and Pepper (S &P) noise. Gaussian blur is incorporated with a Gaussian kernel which has a window size of $7 \times 7$ and variance ranging from 0.1 to 2.0 (0.1, 1.0, 2.0). Random motion blur is applied by using the TorchIO deep learning library [26].

### Training and evaluation

The weights of the ResNet-50 are initialised with Kaiming initialisation and trained for 100 epochs using Adam optimisation (weight decay = 0.01) with a base learning rate of 0.001 [27]. We train the vision transformer and hybrid transformer model from scratch using AdamW optimisation (weight decay = 0.05) with a cosine annealing learning rate scheduler (learning rate = 0.001) for 200 epochs with 10 warm-up epochs.

The convolutional weights in the VQ-VAE are initialised with Kaiming initialisation. The VQ-VAE is trained for 200 epochs using Adam optimisation (weight decay = 0.01) with a base learning rate of 0.0005 [27]. The transformer-based model in stage 2 is trained using AdamW optimisation (weight decay = 0.05) with a cosine annealing learning rate scheduler (learning rate = 0.0001) for 200 epochs with 20 warm-up epochs. We initialise weights in stage 2 of the model with truncated normal initialisation.

Results are evaluated with the accuracy, specificity, precision, recall and AUC. We calculate the specificity, precision, recall and AUC for each class in this 3 class classification problem as a binary classification problem such that we calculate the scores for the group of interest against the other two groups combined. We finally calculate the relative corruption error in the perturbation experiments which is the average change in the AUC performance across all perturbations of our model relative to the models we compare to.

## Results

### Single-domain generalisation

In this set of experiments, we compare our method to BigAug [6], AdvProst [23] and Jigen [24] used to improve the domain generalisability of 3 different deep learning models under an acquisition shift.

**Table 4:** Comparison of 3 different model architectures with 3 different SDG methods with our discrete representation approach (Ours). We evaluate performance with 5 evaluation metrics ± standard deviation

Discrete representations					
	Accuracy	Specificity	Precision	Recall	AUC
Ours	$\mathbf {0.731\pm 0.028}$	$\mathbf {0.720\pm 0.028}$	$\mathbf {0.727\pm 0.046}$	$\mathbf {0.736\pm 0.049}$	$\mathbf {0.739\pm 0.041}$
BigAug[SPAN] [6]					
ResNet-50	$0.720\pm 0.073$	$0.701\pm 0.078$	$0.719\pm 0.055$	$0.724\pm 0.047$	$0.724\pm 0.053$
Hybrid vision transformer	$0.726\pm 0.059$	$0.726\pm 0.033$	$0.720\pm 0.059$	$0.730\pm 0.047$	$0.731\pm 0.060$
3D Vision transformer	$0.646\pm 0.083$	$0.663\pm 0.092$	$0.622\pm 0.082$	$0.648\pm 0.090$	$0.641\pm 0.087$
ProstAdv[SPAN] [23]					
ResNet-50	$0.717\pm 0.066$	$0.708\pm 0.069$	$0.721\pm 0.068$	$0.729\pm 0.070$	$0.730\pm 0.057$
Hybrid vision transformer	$0.722\pm 0.049$	$0.711\pm 0.045$	$0.726\pm 0.061$	$0.729\pm 0.047$	$0.729\pm 0.062$
Vision transformer	$0.620\pm 0.064$	$0.631\pm 0.087$	$0.618\pm 0.077$	$0.633\pm 0.083$	$0.625\pm 0.082$
Jigen[SPAN] [24]					
ResNet-50	$0.690\pm 0.052$	$0.678\pm 0.073$	$0.691\pm 0.088$	$0.696\pm 0.070$	$0.699\pm 0.062$
Hybrid vision transformer	$0.701\pm 0.079$	$0.683\pm 0.099$	$0.702\pm 0.089$	$0.695\pm 0.082$	$0.704\pm 0.079$
Vision transformer	$0.600\pm 0.103$	$0.595\pm 0.117$	$0.606\pm 0.092$	$0.610\pm 0.096$	$0.608\pm 0.094$

Bold indicates the highest score for metric measured

We show the results averaged for the 1.5 T to 3 T and 3 T to 1.5 T domain shift experiments. The metrics were averaged across the three classes

**Fig. 3:**
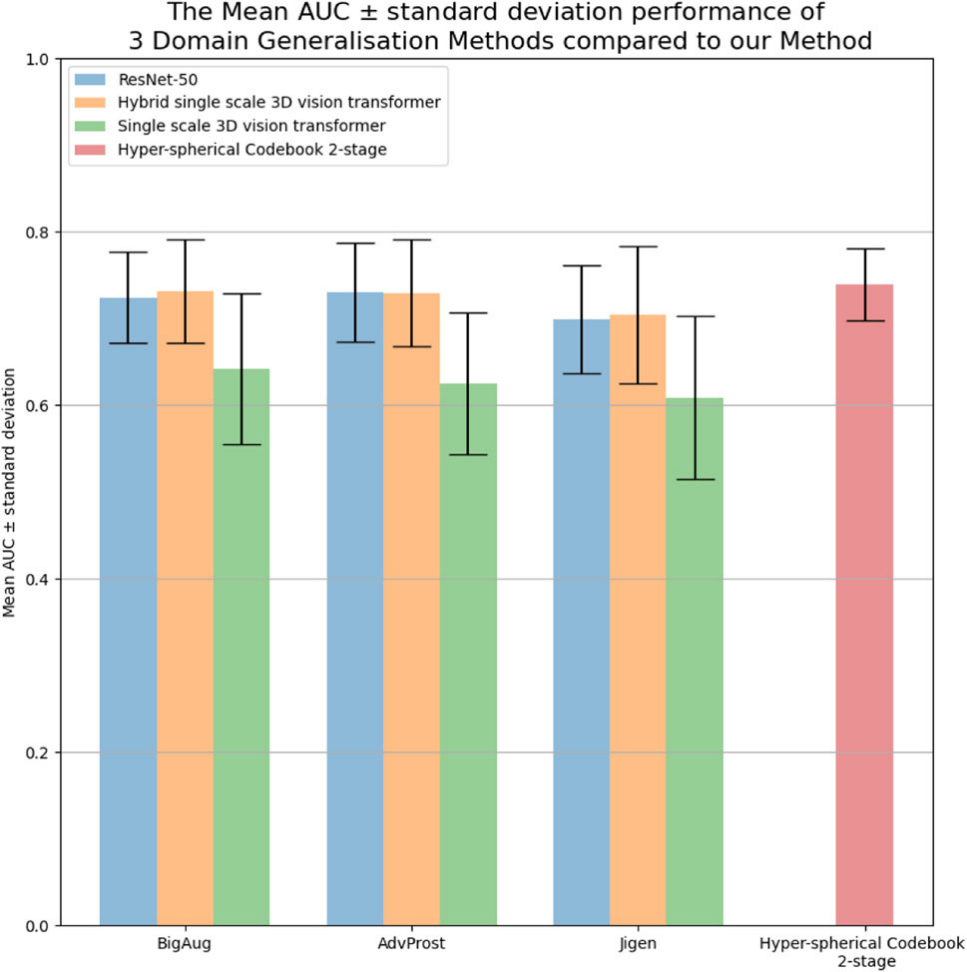
Bar chart comparing AUC performance of 3 domain generalisation applied 3 different models compared to our model without any domain generalisation method applied

The results in Table 4 demonstrate that under an acquisition shift our approach outperforms all 3 of the different domain generalisation methods (augmentation, adversarial and self-supervised methods) applied to a convolutional architecture (ResNet-50), a hybrid convolutional/transformer model (Hybrid 3D vision transformer) and a transformer only model (3D vision transformer) for all evaluation metrics. For example, the AUC score for our method is 0.739 compared to the AUC score of 0.731 obtained by an aggressive augmentation-based method applied to our hybrid model which was the second-best method. Among the domain generalisation methods we compared to, the augmentation-based method (BigAug) obtained the highest AUC score followed by the adversarial method (AdvProst) and then the self-supervised technique (Jigen). Furthermore, among different architectures we compared to, the hybrid architectures overall outperform the ResNet-50 and vision transformer under different SDG methods. For example, under the aggressive augmentation scheme of BigAug, the hybrid vision transformer outperforms the ResNet-50 and vision transformer by 0.007 and 0.09 AUC points, respectively. We further summarise the mean AUC scores and standard deviation obtained by different methods in a bar chart shown in Fig. 3.

### Perturbation experiments

**Table 5:** Mean AUC ± standard deviation for each risk group comparing 4 models types to our model under various perturbations in the input space. The baseline refers to no perturbations applied

	Baseline	Gauss	Poisson	S &P	Blur	Motion
	Low-risk group					
ResNet-50	$0.718\pm 0.074$	$0.665\pm 0.101$	$0.689\pm 0.092$	$0.680\pm 0.076$	$0.698\pm 0.086$	$0.664\pm 0.113$
Hybrid vision transformer	$0.750\pm 0.046$	$0.708\pm 0.090$	$0.708\pm 0.083$	$0.698\pm 0.090$	$0.714\pm 0.084$	$0.685\pm 0.097$
Vision transformer	$0.666\pm 0.086$	$0.616\pm 0.068$	$0.632\pm 0.064$	$0.616\pm 0.090$	$0.630\pm 0.074$	$0.624\pm 0.074$
Ours	$0.777\pm 0.068$	$\mathbf {0.764\pm 0.061}$	$\mathbf {0.758\pm 0.083}$	$\mathbf {0.754\pm 0.052}$	$\mathbf {0.770\pm 0.071}$	$\mathbf {0.740\pm 0.084}$
	Medium risk group					
ResNet-50	$0.732\pm 0.084$	$0.680\pm 0.114$	$0.694\pm 0.113$	$0.696\pm 0.083$	$0.699\pm 0.090$	$0.681\pm 0.113$
Hybrid vision transformer	$0.773\pm 0.054$	$0.724\pm 0.090$	$0.706\pm 0.114$	$0.710\pm 0.084$	$0.727\pm 0.098$	$0.695\pm 0.104$
Vision transformer	$0.670\pm 0.083$	$0.620\pm 0.075$	$0.636\pm 0.054$	$0.621\pm 0.078$	$0.641\pm 0.065$	$0.609\pm 0.079$
Ours	$0.781\pm 0.075$	$\mathbf {0.770\pm 0.061}$	$\mathbf {0.761\pm 0.084}$	$\mathbf {0.759\pm 0.054}$	$\mathbf {0.768\pm 0.074}$	$\mathbf {0.742\pm 0.085}$
	High risk group					
ResNet-50	$0.740\pm 0.090$	$0.694\pm 0.118$	$0.704\pm 0.114$	$0.708\pm 0.080$	$0.710\pm 0.093$	$0.688\pm 0.124$
Hybrid vision transformer	$0.781\pm 0.068$	$0.729\pm 0.094$	$0.721\pm 0.118$	$0.728\pm 0.093$	$0.741\pm 0.134$	$0.694\pm 0.116$
Vision transformer	$0.671\pm 0.093$	$0.636\pm 0.071$	$0.633\pm 0.051$	$0.628\pm 0.081$	$0.646\pm 0.064$	$0.609\pm 0.083$
Ours	$0.785\pm 0.071$	$\mathbf {0.770\pm 0.069}$	$\mathbf {0.762\pm 0.081}$	$\mathbf {0.759\pm 0.054}$	$\mathbf {0.775\pm 0.077}$	$\mathbf {0.747\pm 0.084}$

Bold indicates the highest AUC score under each perturbation

The results shown are averaged across all perturbation parameters, i.e. overall noise levels for Gaussian, Poisson and Salt and Pepper (S &P) noise

**Table 6:** Relative corruption error (%) based on AUC of our model relative to the 3 other models

	Low risk	Medium risk	High risk
ResNet-50	51.0	50.0	57.1
Hybrid vision transformer	41.8	34.6	38.3
Vision transformer	46.7	47.1	55.1

The results in Table 5 demonstrate that by using our method, the AUC score diminishes far less under various textural and spatial perturbations compared to the other models. This is true for all risk groups as highlighted in bold in Table 5. For example, the AUC score for our approach only diminishes by only 1.2 points on average across all risk groups under Gaussian noise averaged across all noise. This is compared to the AUC decreasing by 5.0, 4.8 and 4.5 points for the ResNet-50, hybrid vision transformer and vision transformer, respectively, under Gaussian noise. This shows how discrete representations as input into a transformer architecture can significantly improve the robustness to textural perturbations. In this example, it appears that the attention-based methods in the form of the vision transformer and hybrid method are more robust to noise compared to the convolutional-only architecture. In regard to the spatial perturbations, our approach only diminishes by 3.8 points on average across all risk groups under motion artefact compared to by 5.2, 7.7 and 6.0 points for the ResNet-50, hybrid vision transformer and vision transformer, respectively. Here, we notice the opposite trend compared to the noise-based perturbations where the convolutional architectures outperform the vision transformer and hybrid method under spatial perturbations. The improved robustness under spatial perturbations of CNNs compared to the transformer-based architecture might well arise from the loss of positional information which is key to the transformer architecture. It has been shown that MHSA demonstrates exceptional robustness specifically against high-frequency noise [28, 29] compared to convolutions which might explain the hybrid network and vision transformer outperforming the ResNet-50 under noise-based perturbations. This is because MHSA and convolutions display contrasting characteristics. MHSA aggregates feature maps by ensembling, whereas convolutions differentiate them [29]. Furthermore, a Fourier analysis of the feature maps reveals that MHSA suppresses high-frequency signals, while convolutions amplify high-frequency elements [29]. This means that the MHSA function acts as low-pass filter, while convolutions serve as high-pass filters. Additionally, this makes convolutions susceptible to high-frequency noise, while MHSA remains unaffected [29].

Finally, in Table 6, we show that the relative corruption error in terms of the AUC of our model relative to the 3 other models in Table 5 is significantly less than 1 for all 3 risks groups. This shows our model demonstrates superior robustness performance averaged across all types of input perturbations compared to the 3 model architectures in Table 6. For example, we show the relative corruption error of the ResNet-50 relative to our method is 51.0, 50.0 and 57.1 for the low-, medium- and high-risk groups, respectively. Among different architectures, it appears the ResNet-50 is the most robust compared to the vision transformer and hybrid architecture. This shows that the convolutional-based architecture is more robust than the attention-based methods when averaged across all different perturbations. The overall improved performance of the Resnet-50 arises from its superior robustness to spatial perturbations.

## Conclusion

In conclusion, we show that we can improve the robustness of the vision transformer under an acquisition shift from 1.5 T to 3 T and vice versa using a discrete input obtained from the vector-quantised high abstraction CNN features in the latent space of a CNN auto-encoder. We apply our method in the task of prostate disease classification and outperform various CNN-, transformer- and hybrid- based models achieving an AUC score of 0.739. We also show that our approach outperforms augmentation-based, adversarial and self-supervised methods in terms of all the evaluation metrics used. We finally show how our method is robust under various spatial and texture-based perturbations achieving a relative corruption error in terms of the AUC significantly less than 1 compared to various deep learning architectures.
